# Low circulatory Fe and Se levels with a higher IL-6/IL-10 ratio provide nutritional immunity in tuberculosis

**DOI:** 10.3389/fimmu.2022.985538

**Published:** 2023-01-12

**Authors:** Sandeep R. Kaushik, Sukanya Sahu, Hritusree Guha, Sourav Saha, Ranjit Das, Rukuwe-u Kupa, Wetetsho Kapfo, Trinayan Deka, Rumi Basumatary, Asunu Thong, Arunabha Dasgupta, Bidhan Goswami, Amit Kumar Pandey, Lahari Saikia, Vinotsole Khamo, Anjan Das, Ranjan Kumar Nanda

**Affiliations:** ^1^ Translational Health Group, International Centre for Genetic Engineering and Biotechnology, New Delhi, India; ^2^ Department of Respiratory Medicine, Agartala Government Medical College, Agartala, Tripura, India; ^3^ Healthcare Laboratory and Research Centre, Naga Hospital Authority, Kohima, Nagaland, India; ^4^ Department of Microbiology, Assam Medical College, Dibrugarh, Assam, India; ^5^ District Tuberculosis Centre, Kohima, Nagaland, India; ^6^ Department of Medicine, Agartala Government Medical College, Agartala, Tripura, India; ^7^ Department of Microbiology, Agartala Government Medical College, Agartala, Tripura, India; ^8^ Mycobacterial Pathogenesis Laboratory, Translational Health Science and Technology Institute (THSTI), Faridabad, Haryana, India; ^9^ Department of Microbiology, Gauhati Medical College, Guwahati, Assam, India

**Keywords:** iron metabolism, tuberculosis, anemia of inflammatiom, cytokines, selenium

## Abstract

Tuberculosis (TB) patients show dysregulated immunity, iron metabolism, and anemia. In this study, circulatory cytokines, trace metals, and iron-related proteins (hepcidin, ferroportin, transferrin, Dmt1, Nramp1, ferritin, ceruloplasmin, hemojuvelin, aconitase, and transferrin receptor) were monitored in case (active tuberculosis patients: ATB) and control (non-tuberculosis: NTB and healthy) study populations (*n* = 72, male: 100%, mean age, 42.94 years; range, 17–83 years). Using serum elemental and cytokine levels, a partial least square discriminate analysis model (PLS-DA) was built, which clustered ATB patients away from NTB and healthy controls. Based on the PLS-DA variable importance in projection (VIP) score and analysis of variance (ANOVA), 13 variables were selected as important biosignatures [IL-18, IL-10, IL-13, IFN-γ, TNF-α, IL-5, IL-12 (p70), IL-1β, copper, zinc, selenium, iron, and aluminum]. Interestingly, low iron and selenium levels and high copper and aluminum levels were observed in ATB subjects. Low circulatory levels of transferrin, ferroportin, and hemojuvelin with higher ferritin and ceruloplasmin levels observed in ATB subjects demonstrate an altered iron metabolism, which partially resolved upon 6 months of anti-TB therapy. The identified biosignature in TB patients demonstrated perturbed iron homeostasis with anemia of inflammation, which could be useful targets for the development of host-directed adjunct therapeutics.

## Introduction

Tuberculosis (TB) is caused by *Mycobacterium tuberculosis* (*Mtb*) infection and is still a major killer worldwide (1). TB patients present with a dysregulated immune system and release pro-inflammatory cytokines inducing a cascade of activities hampering iron homeostasis (2). Hepcidin from hepatocytes is released into the circulation, which subsequently binds to ferroportin, the only known iron exporter, and induces its degradation through ubiquitylation (3). Increased ferroportin degradation leads to reduced circulatory iron and causes anemia of inflammation (AI) (2, 4). Furthermore, during AI, even though the host may have enough stored iron, it becomes unavailable for use and the dietary iron absorption is lowered as well. Although the AI is described as a hallmark of TB, it is only present in a subset of TB patients as shown by a Korean study (5). Moreover, as the metabolic crossroads of iron are also linked to copper, zinc, and selenium levels, and due to their heightened reactivity, intracellular levels of these trace metals are regulated critically by the host (6, 7). However, the host system exploits the indispensability and toxicity of these metals to safeguard itself from bacterial invaders (8). Nutritional immunity is involved in the mechanism that withholds trace metals like iron to limit the pathogen’s growth (9, 10). The iron carrier protein transferrin also plays a major role in nutritional immunity by binding and removing labile iron from circulation. In addition, iron transporters Nramp1 and Nramp2/Dmt1 play an essential part in maintaining intracellular iron levels. Nramp2/Dmt1 also helps the host in the dietary uptake of iron from the duodenum. Interlinking host immuno-profile with levels of circulatory trace metals and iron-related proteins may help to better understand the pathophysiology of TB and design newer approaches to develop adjunct therapeutics for TB. In this study, these important molecules were monitored in newly diagnosed TB patients, controls, and followed up TB patients.

## Methodology

### Ethics statement and subject recruitment

**Figure 1:**
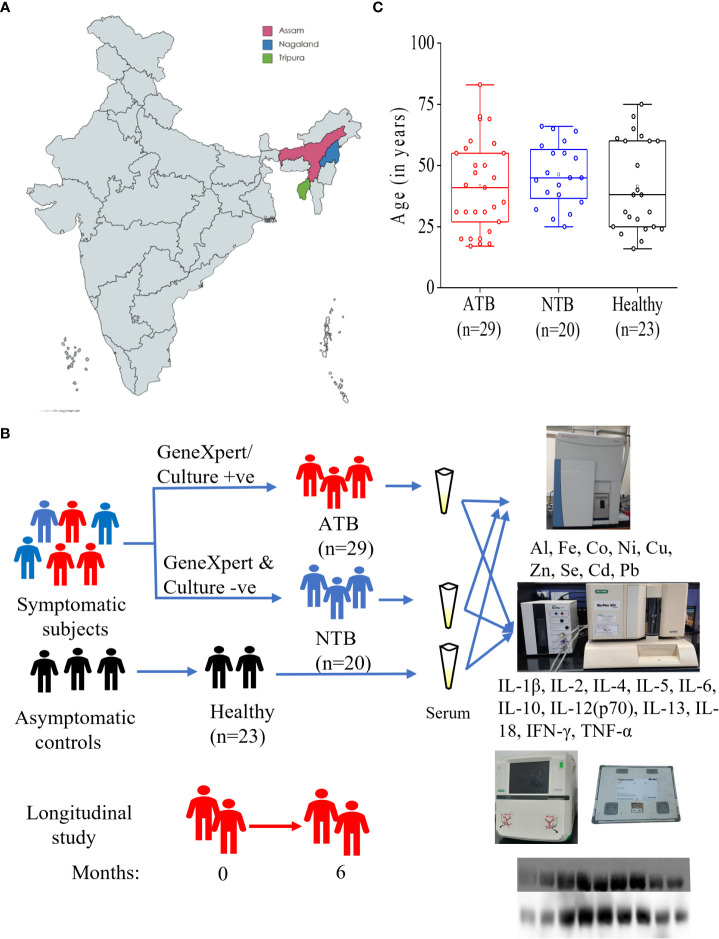
Schematic representation of study cites **(A)**, subject classification for serum elemental, cytokine and iron metabolism related protein analyses **(B)**. Box plot representing the age of active tuberculosis (ATB), non-tuberculosis (NTB) and healthy control subjects **(C)**.

This study was part of a project activity approved by the institutional review board and ethics committees of Agartala Government Medical College-Agartala (protocolF.4[6-9]/AGMC/Academic/IEC Committee/2015/8965, dated 25 April 2018), Nagaland Hospital Authority-Kohima (NHAK/HLRC/RES3/2013/64), Assam Medical College-Dibrugarh (AMC/EC/PG/3530), and International Centre for Genetic Engineering and Biotechnology (ICGEB) New Delhi (ICGEB/IEC/2014/07). After receiving signed informed consent, subjects presenting with 2 weeks of cough, fever, and weight loss at the outpatient departments of the partnering hospitals were recruited (**Figure 1A**).

### Sample collection and subject classification

The sputum and whole blood samples (for serum preparation) were collected from the study subjects. Collected sputum samples were subjected to microscopy and GeneXpert tests, and subjects with positive test results for both tests were grouped as active tuberculosis patients (ATB) while those with negative results were grouped as non-TB (NTB) (**Figure 1B**) (11). NTB subjects were clinically diagnosed as suffering from asthma, chronic obstructive pulmonary diseases (COPD), lung cancer, and pneumonia. Only male subjects with negative HIV test results were selected. Healthy subjects from a similar background of patients who did not receive any medication for at least 1 week before sampling were recruited as controls. Whole blood samples (4 ml) were collected in vacutainer tubes for serum isolation by centrifuging at 1,500 *g* for 10 min at 4°C. Aliquots of serum were stored at −80°C till further analysis and a maximum of two freeze–thaw cycles were allowed. For the longitudinal study, TB patients were followed up until completion of 6 months of anti-TB treatment [recommended by Revised National TB Control and Programme (RNTCP), Government of India]. TB patients were given a fixed dose of four drugs (isoniazid, rifampicin, ethambutol, and pyrazinamide) during 2 months of intensive phase followed by two drugs (isoniazid and rifampicin) during the continuation phase (**Figure 1B**).

### Micronutrient profiling in serum samples using inductive coupled plasma mass spectrometry (ICP-MS)

Serum samples (50 µl) were subjected to microwave digestion using HNO_3_ (225711, Sigma, USA) and H_2_O_2_ (Supelco, 107298, Hydrogen peroxide 30% Suprapur^®^) in a Multiwave-Pro digestor (Anton Paar, USA) for 30 min at 140°C. The digested serum samples were diluted using ultrapure water (Honeywell) to bring the acid concentration below permissible limit for ICP-MS data acquisition. The diluted digested samples were fed to ICP-MS (Thermo Scientific iCAP-TQ) in kinetic energy discrimination (KED) mode using helium for data acquisition (12, 13). Serum trace metal levels in the test samples were quantified using the total dilution factor.

### Serum cytokine profiling

Serum pro-inflammatory [interleukin (IL)-1β, IL-18, IL-2, IL-6, IL-12, interferon (IFN)-γ, and tumor necrosis factor (TNF)-α] and anti-inflammatory cytokine (IL-4, IL-5, IL-10, and IL-13) levels were quantified using a Bioplex Microplate array reader (Bio-Rad Bio-Plex 200 Systems, USA) by using a 11-plex custom kit from Bio-Rad. Briefly, to the diluted serum samples, conjugated beads (Bio-Rad, USA) were added followed by biotinylated detection antibodies. After adding streptavidin to the test samples, standards, and blanks, the fluorescence intensity for all the bead regions was measured using a Bioplex Microplate array reader (Bio-Rad Bio-Plex 200 Systems, USA). All the washing steps were performed using the MAGPIX wash station.

### Western blot experiment

Equal amounts of serum proteins related to iron metabolism (transferrin, transferrin receptor, ferroportin, hepcidin, Dmt1, Nramp1, aconitase, hemojuvelin, ferritin, and ceruloplasmin) from study subjects were probed by Western blot analyses. Briefly, denatured serum proteins were loaded on sodium dodecyl sulfate-polyacrylamide gel electrophoresis (SDS-PAGE) gel for separation and transferred to PVDF membrane using a Semi-dry transfer apparatus (TE77, semi dry apparatus, Amersham). The blots were incubated with primary antibody (transferrin; ab82411, dilution: 1/10,000), transferrin receptor; ab1086 (1/500), ferroportin; ab78066 (1/500), hepcidin; ab30760 (1/250), Dmt1; ab55735 (1/500), Nramp1; ab59696 (1/500), aconitase; ab126595 (1/1,500), and hemojuvelin; ab54431 (1/1,000) from Abcam, USA and ferritin; D1D4 (1/1,000) and ceruloplasmin; D7Q5W (1/1,000) from Cell Signaling Technology, USA) overnight at 4°C using a shaker. After washing, the blots were incubated with secondary antibody (Anti-Rabbit, Sigma A-6154) for 2 h and developed using Pico-Plus enhanced chemiluminescence (ECL) (Pierce, Thermo Fisher) on x-ray films or imaging system (ChemiDoc MP Bio-Rad, USA). ImageJ (version 1.53e) was used for densitometric calculations. Parallel gels were run using an equal serum protein amount (5 µg) from the study samples and silver stained for every blot.

### Statistical analysis

MetaboAnalyst 5.0 tool was used for Partial Least Square-Discriminate Analysis (PLS-DA) model building (14). Variables with a variable importance in projection (VIP) score >0.6 and analysis of variance (ANOVA) were selected as important markers. OriginPro 2020b (64-bit) 9.7.5.184 (Student Version) was used for box plots, line plots, scatter plots, and correlation analysis. Univariate statistical tools like Student’s *t*-test (paired or unpaired) were performed to calculate the significance level of variation between groups and a *p*-value <0.05 was considered as statistically significant at the 95% confidence interval.

## Results

### Study subjects

A total of 72 male subjects from northeastern parts of India were included in this study (**Figure 1A** and **Table 1**). Circulatory iron levels are significantly altered during the reproductive cycle of female subjects and thus excluded from the present study (4, 15). All study subjects were age-matched including ATB (*n* = 29, mean age in years 41.62), NTB (*n* = 20, 46.30), and healthy (*n* = 23, 41.43) groups and show statistically insignificant variation in their age between study groups (**Figure 1C**). Approximately 40% of these patients were smokers or ex-smokers in ATB and NTB groups (**Table 1**).

**Table 1:** Epidemiological details of the study subjects used in this study.

Study groups	Total	ATB	NTB	Healthy
Subject (*n*)	72	29	20	23
Clinical sites (AGMC/AMC/NHAK)	46/14/12	15/8/6	14/3/3	17/3/3
Mean age (range) in years	42.94 (16–83)	41.62 (17–83)	46.30 (25–66)	41.43 (16–75)
Male (%)	100%	100%	100%	100%
AFB or GeneXpert (+ve/−ve/na)	29/20/23	29/-/-	-/20/-	-/-/23
Alcoholic (yes/no/Ex/na)	12/38/1/21	1/16/-/12	4/12/1/3	7/10/-/6
Smoker (yes/no/Ex/na)	20/28/3/21	4/11/2/12	7/9/1/3	9/8/-/6
Expectoration (yes/no/na)	41/14/17	23/5/1	15/2/3	3/7/13
Cough (yes/no/na)	49/6/17	25/3/1	17/-/3	7/3/13
Fever (yes/no/na)	19/33/18	13/15/2	6/8/3	-/10/13
Hemoptysis (yes/no/na)	10/46/16	7/22/-	3/14/3	-/10/13
Chest Pain (yes/no/na)	24/31/17	15/13/1	6/11/3	3/7/13
Breathlessness (yes/no/na)	21/33/17	13/14/1	6/11/3	2/8/13
Wheeze (yes/no/na)	15/38/19	9/18/2	4/12/4	2/8/13

AGMC, Agartala Government Medical College Agartala; AMC, Assam Medical College-Dibrugarh; NHAK, Nagaland Hospital Authority-Kohima; +ve, positive; −ve, negative; na, Not available; n, sample size (available data).

### Tuberculosis patients show reduced circulatory iron and selenium levels

**Figure 2:**
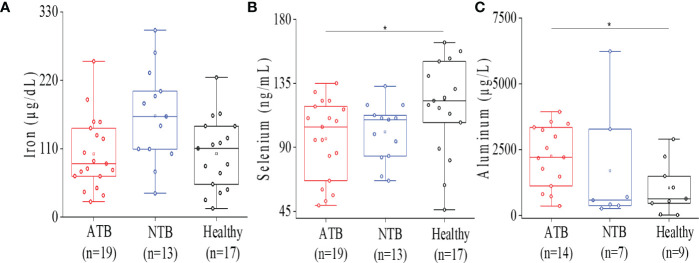
Serum ICP-MS analysis showed altered micro elemental profile in ATB patients. Box plot representation for iron levels in ATB, NTB, and healthy groups **(A)**. Box plot representation for selenium levels in ATB, NTB, and healthy groups **(B)**. Box plot representation for aluminum levels in ATB, NTB, and healthy groups **(C)** (*p < 0.05).

Lower levels of circulatory iron were observed in the ATB study group compared to healthy controls (**Figure 2A**). Although the serum iron levels were quite low in all ATB subjects, it was not statistically significant compared to healthy subjects. In ATB subjects, lower selenium levels were observed compared to healthy subjects (**Figure 2B**). Additionally, lower selenium levels were observed in NTB subjects as well compared to healthy individuals (**Figure 2B**). Interestingly, both TB patients and disease control groups showed similar selenium levels, and it was found to be significantly lower only in the ATB group compared to healthy controls group (*p* < 0.05).

### Higher aluminum abundance can be a risk factor for TB

Higher aluminum levels were noticed in ATB patients consistently compared to both control groups (**Figure 2C**). Aluminum levels were found to be elevated (>2-fold) in the ATB group (*p* < 0.05) compared to the healthy group. Interestingly, both ATB and NTB patient groups showed higher serum aluminum levels compared to healthy controls (**Figure 2C**).

### Pro-inflammatory cytokines were comparable except IL-18

**Figure 3:**
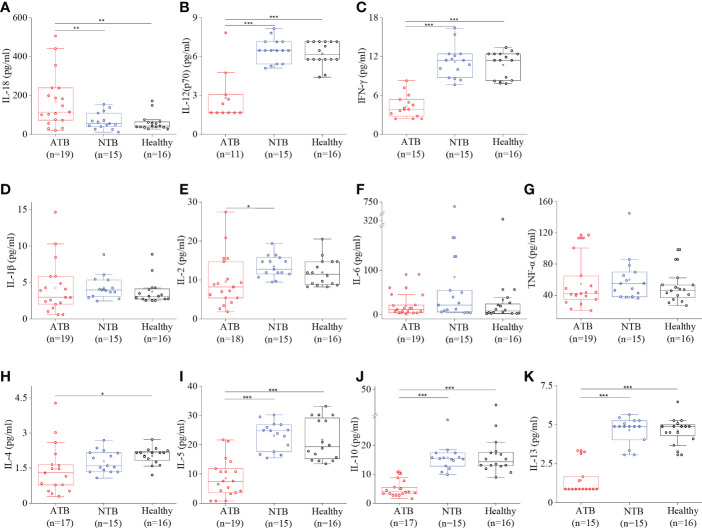
Cytokine profiling showed a robust Th1 response in ATB patients. Box plots for individual serum cytokine levels using Bioplex-200 for **(A)** IL-18, **(B)** IL-12, **(C)** IFN-γ, **(D)** IL-1β, **(E)** IL-2, **(F)** IL-6, **(G)** TNF-α, **(H)** IL-4, **(I)** IL-5, **(J)** IL-10, and **(K)** IL-13 in ATB, NTB, and healthy groups (*p < 0.05, **p < 0.01, ***p < 0.001).

High circulatory interleukin-18 (IL-18) levels were observed in ATB compared to both NTB and healthy controls (*p* < 0.01) (**Figure 3A**). The IL-18 levels were roughly threefold higher in the ATB group compared to the NTB group (fold change: FC = 2.46) and healthy group (FC = 2.74). IL-1β, IL-2, IL-6, and TNF-α levels were comparable among all three groups (**Figures 3D–G**). Interestingly, we observed lower IL-12 and IFN-γ levels in the ATB group (**Figures 3B, C**).

### Cytokine profiling revealed downregulated Th2 cytokines’ expression

A lower level of anti-inflammatory cytokines (IL-4, IL-5, IL-10, and IL-13) was observed in the ATB group compared to healthy controls (**Figures 3H–K**). The anti-inflammatory interleukins were found to be comparable in both control groups and the lowest in the ATB group. It shows an overall low Th2 cytokines’ profile in the ATB group.

### IL-6/IL-10 and TNF-α/IL-10 ratios characterized the ATB profile

**Figure 4:**
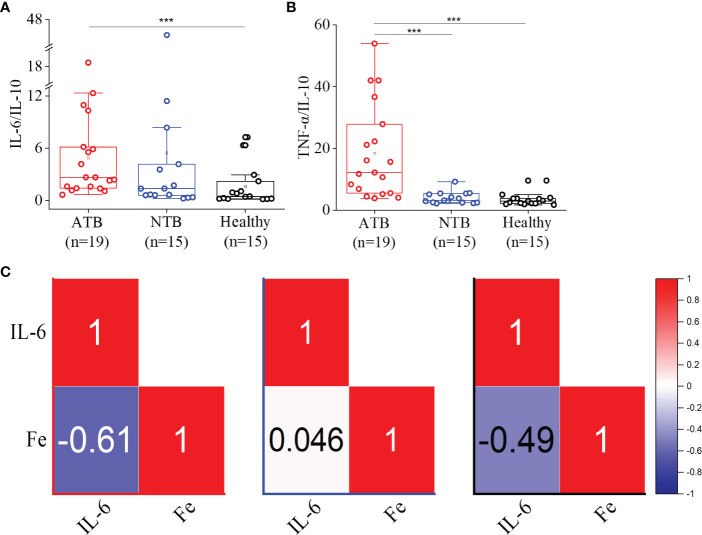
Higher IL-6/IL-10 and TNF-α/IL-10 ratios observed in ATB patients. Box plots for IL-6/IL-10 **(A)** and TNFα/IL-10 **(B)** ratios in the ATB, NTB, and healthy study groups. Correlation (Pearson *r*) values between the serum IL-6 and iron (Fe) levels in active tuberculosis (ATB), non-tuberculosis (NTB), and healthy control groups **(C)** (***p < 0.001).

Although the IL-6 levels were comparable between three groups, a significantly higher IL-6/IL-10 were observed in the ATB study group (threefold) compared to the healthy group (4.88, 1.58; *p* < 0.05) (**Figure 4A**). Similarly, we observed a more than fivefold higher TNF-α/IL-10 in the ATB group (18.4, *p* < 0.001) compared to the healthy group (3.4) (**Figure 4B**). This indicated Th1 cytokine predominance and pronounced Th1 expression in the ATB group. Interestingly, we noticed a strong inverse correlation between IL-6 and iron levels in the ATB group, which was absent in the NTB group (**Figures 4C**, **S1**).

### A potential biosignature of essential elements and cytokines could differentiate ATB patients from controls

**Figure 5:**
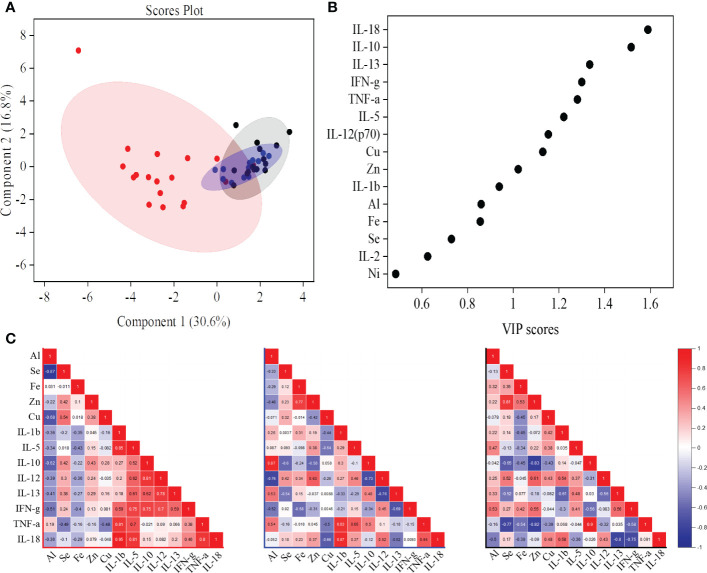
A partial least squares discriminant analysis (PLS-DA) model of identified serum cytokines and trace metals showed the active tuberculosis (ATB) study group cluster away from non-tuberculosis (NTB) and healthy control subjects **(A)**. Important cytokines and trace metals identified based on the variable importance in projection (VIP) score from the PLS-DA model **(B)**. Correlation (Pearson *r*) values between important markers [common molecules from VIP score and analysis of variance (ANOVA)] in ATB, NTB, and healthy study groups **(C)**.

We combined the cytokine and trace metal data to build a predictive partial least square discriminate analysis (PLS-DA) model that could cluster the ATB study subjects away from both control groups (**Figure 2**, **S2**, **3**, **5A**). Based on the VIP score and analysis of variance (ANOVA), a total of 13 variables [IL-18, IL-10, IL-13, IFN-γ, TNF-α, IL-5, IL-12 (p70), IL-1β, Cu, Zn, Al, Fe, and Se] were selected for pairwise correlation (Pearson *r*) analysis (**Figures 5B, C**). The IL-1β, IFN-γ, TNF-α, and IL-18 cytokines were found to be highly correlated with each other in the ATB group. In addition, a highly negative correlation (*r* = −0.87) was observed between selenium and aluminum in ATB patients (**Figures 5C**, **S3**). Furthermore, we observed a fairly negative correlation between IFN-γ and aluminum in ATB (*r* = −0.5) and NTB (*r* = −0.52), while a moderately positive correlation was observed in the healthy control group (*r* = 0.53) (**Figures 5C**, **S3**). Also, we noticed a strong correlation (*r* = 0.87) between aluminum and IL-10 in the NTB group (**Figures 5C**, **S3**). In addition, iron, zinc, and selenium were also found to be fairly correlated among themselves, while a weak to moderate correlation was observed with certain cytokines (**Figures 5C**, **S3)**.

### Higher expression of inflammatory marker proteins observed in ATB and NTB subjects

**Figure 6:**
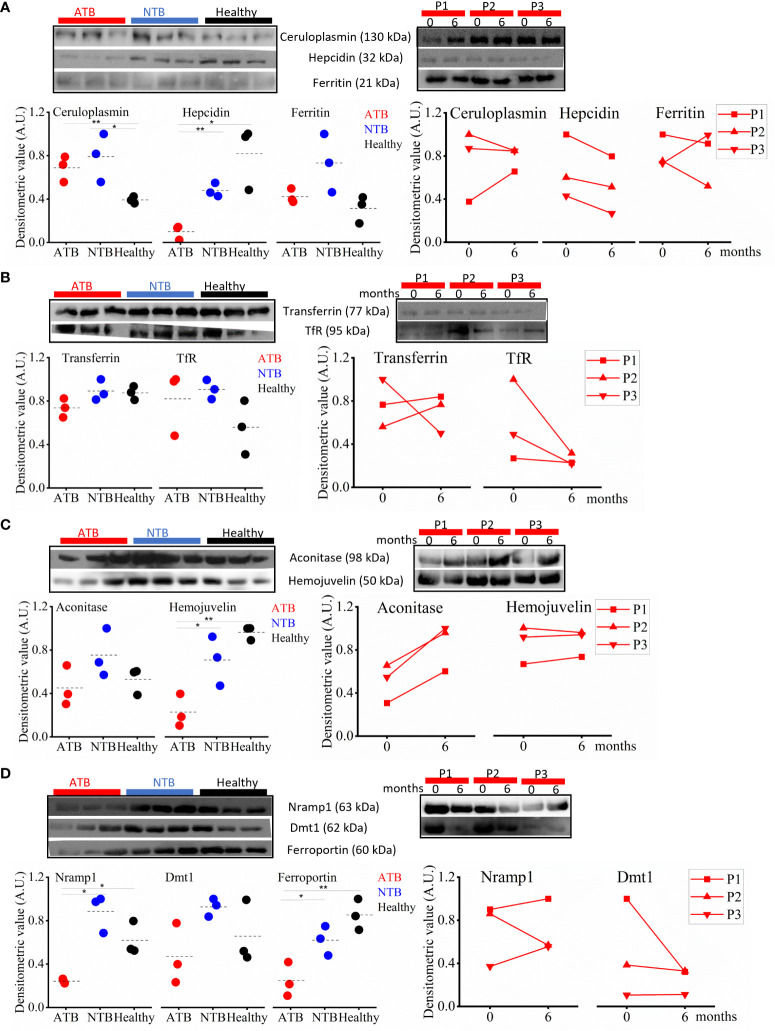
Perturbed iron homeostasis indicating iron sequestration observed in active tuberculosis (ATB) patients. Western blot images and densitometric value expression in ATB, non-tuberculosis (NTB), and healthy controls and in longitudinally followed up tuberculosis patients at case presentation (0 months) and upon completion of treatment (6 months) in inflammatory marker proteins (ceruloplasmin, ferritin, and hepcidin) **(A)**, iron carrier proteins (transferrin and TfR) **(B)**, iron-sensing proteins (hemojuvelin and IRP1) **(C)**, and iron transporter proteins (ferroportin1, Nramp1, and Dmt1) **(D)** (P, patient; TfR, Transferrin receptor; IRP1, Iron regulatory protein 1; Nramp1, Natural resistance-associated macrophage protein 1; Dmt1, Divalent metal ion transporter 1; A.U., arbitrary unit; *p < 0.05; **p < 0.01).

In chronic diseases, like TB, higher levels of inflammatory marker proteins (ceruloplasmin and ferritin) were expected, and we also observed higher abundance in ATB patients and NTB controls compared to healthy subjects (**Figure 6A**). In addition, we also monitored the hepcidin levels, and the lowest levels were observed in the ATB group. In longitudinally followed up ATB patients, we observed a treatment-induced reduction in the abundance of these proteins (**Figure 6A**).

### Iron carrier proteins were found to be reduced in TB patients

The iron carrier protein (i.e., transferrin) levels were monitored in serum samples of study subjects to get a better understanding of iron homeostasis. Interestingly, the serum transferrin levels in ATB patients were lower compared to both control groups (**Figure 6B**). Interestingly, the circulatory sTfR (serum transferrin receptor) levels were higher in TB patients compared to healthy controls (**Figure 6B**). Both these proteins showed a reverse longitudinal trend in followed up patients, and we noticed increased transferrin levels with reduced TfR levels in ATB patients completing 6 months of TB treatment compared to their drug-naïve status (**Figure 6B**).

### Iron-sensing protein hemojuvelin and aconitase 1 (IRP1) were found altered in TB patients

Furthermore, we monitored the expression of hemojuvelin (HJV), an important iron-sensing protein, and observed a significantly lower circulatory level in ATB patients compared to controls (**Figure 6C**). We also monitored the circulatory level of the iron regulatory protein (IRP1) and observed lower levels of it in ATB subjects compared to controls (**Figure 6C**). In the longitudinally followed samples, the trend of HJV and IRP1 showed significant improvement in patients completing TB treatment (**Figure 6C**).

### Iron transporters and their expression in TB patients

Iron transporters play a critical role in iron metabolism, and we monitored the circulatory levels of iron transporters (ferroportin, Nramp1, and Dmt1) to assess the altered iron metabolism in TB patients. The lowest ferroportin levels were observed in ATB patients, with intermediate levels in NTB patients and the highest levels in healthy subjects (**Figure 6D**). We also monitored circulatory Nramp1 levels and interestingly observed significantly lower levels in ATB patients compared to both control groups, which slightly improves after completion of treatment (**Figure 6D**). A similar trend was also noticed for Nramp2/Dmt1 (**Figure 6D**).

## Discussion

**Figure 7:**
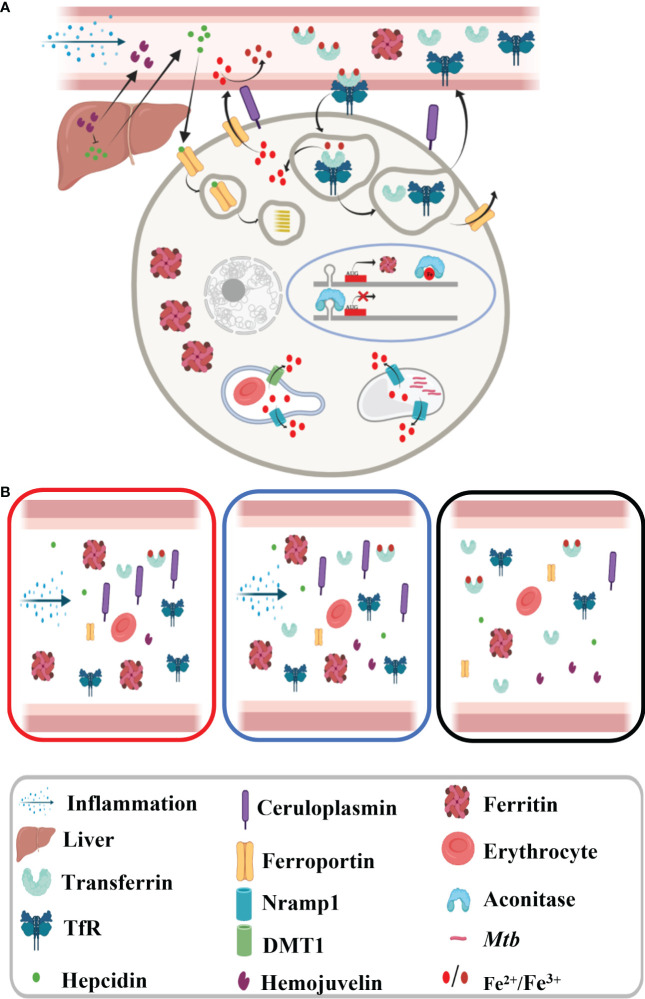
Schematic diagram showing iron metabolism in healthy subjects. **(A)** Macrophage showing iron metabolism homeostasis and **(B)** dysregulated iron metabolism in tuberculosis patients and non-tuberculosis subjects compared to healthy controls.

Iron is an important metal for all life forms as it is involved in several key processes like DNA synthesis, energy production, and respiration; thus, iron homeostasis is extremely vital. Iron homeostasis is regulated by several key hormones and transporters, which modulate the cellular and circulatory iron levels in response to various stimuli such as erythropoiesis requirement, and inflammation and change in labile iron pool constitute an integral part of this modulation (**Figure 7A**) (4). Free iron is known to cause Fenton reaction and is thus involved in free radical generation, which can aggravate the inflammation. Therefore, iron levels are maintained in a narrow range to avoid excess or deficiency, both of which are detrimental to the host.

Iron is equally important to the *Mtb* as to the host, and thus, there is a constant fight for this crucial element between host and pathogen. Humans and *Mtb* have evolved constantly to develop various strategies to control this essential element, and it is evident that the host developed intrinsic tools to keep the available iron in check and limits its access to *Mtb*. From our results and literature, it is proven that the host takes imminent steps to lock the iron in cellular stores even at the cost of self-damage (anemia); thus, there is significantly low iron present in circulation (16). As iron is one of the important redox elements, we have looked into other redox elements as well and noticed that copper levels were marginally increased during infection (**Figure S2**). We noticed a severe drop in selenium levels, which is extremely important for free radical neutralization being part of selenoproteins including glutathione peroxidase (17). Through nutritional immunity, the host sequesters circulatory trace metals, including iron, to limit pathogenicity during infection and the observed changes in trace metal levels might be benefiting the host (9, 10, 15–17).

We have also estimated the serum aluminum levels of study subjects. Interestingly, serum aluminum levels were found to be significantly higher in ATB patients and were inversely correlated to selenium levels (**Figures 2**, **5C**). As we know, aluminum is a trivalent ion, and it can replace ferric iron. Higher circulatory aluminum may saturate transferrin binding sites and aluminum toxicity may impact hematopoiesis under hypoferric conditions (18, 19). Our findings indicate that low iron and high aluminum circulatory levels in TB patients have a negative impact on the immunity. Interestingly, we noticed a negative correlation between aluminum levels and IFN-γ, which could be responsible for lower IFN-γ levels observed in ATB patients. The role of aluminum in TB disease establishment and pathology needs further validation in a larger cohort. In the study population, circulatory levels of other microelements (zinc, nickel, and cobalt) did not show significant variation between the groups (**Figure S2**). In addition, heavy metals like lead and cadmium were monitored, and a marginally higher cadmium level in ATB patients that was below the toxic level (<5 µg/L) (**Figure S2**) was observed (20).

It is well known that in chronic inflammatory conditions, the pro-inflammatory as well as anti-inflammatory cytokine profiles are altered. Inflammation is an integral part of infectious diseases like TB and similar observations were observed in our study subjects (21). IL-18 is a pro-inflammatory cytokine and belongs to the IL-1 superfamily like IL-1β, and we observed a strong correlation between these two (*r* = 0.95). A higher level of circulatory IL-18 was observed in ATB patients corroborating earlier reports (22). It initiates the cascade of signaling to induce Th1 response especially IFN-γ in the presence of IL-12 and enhances the cell-mediated cytotoxicity (23, 24). As expected, IL-18 levels were significantly high in TB patients, but to our surprise, we did not witness a high IL-12 level, which further shows why we did not observe a mounted IFN-γ response in contrast to earlier reports (22, 25). Another reason for low serum IFN-γ levels could be because IFN-producing cells localize near the site of infection in TB, resulting in low circulatory IFN-γ levels. Interestingly, other important pro-inflammatory cytokines like IL-1β, IL-2, IL-6, and TNF-α did not show any significant changes among study groups but were found to be correlated with each other in the ATB group (**Figures 3D–G**). At the same time, we did notice that the anti-inflammatory cytokine IL-4 was significantly low in ATB patients compared to controls. IL-4 determines the Th2 response through STAT6 and GATA3. As IL-4 is low, it is anticipated that Th2 cytokines will be low, and similar findings were observed in this study. Importantly, high IL-6/IL-10 and TNF-α/IL-10 ratios were observed in ATB patients. Lower levels of IFN-γ and IL-10 have been reported to be directly linked to TB cure, and we have observed a positive correlation between these two (26). From the individual cytokine levels, it seems that ATB patients do not show a strong Th1 response, but the cytokine ratios prove it otherwise (**Figures 4A, B**).

The inflammatory response in infectious diseases like TB lowers labile iron pool and alters erythropoiesis requirements, which may be contributed by differential expression of iron carrier, transporters, and storage proteins. First, we looked into the inflammatory marker proteins’ expression and found significantly higher circulatory ceruloplasmin and ferritin levels, and as expected, reduced abundance of these molecules was observed in ATB patients completing anti-TB therapy (**Figures 6A**, **7B**) (16, 27, 28). Ferritin stores iron; hence, under infection-associated inflammation conditions, its level should increase to sequester the iron in cellular stores. Next, we looked at the hepcidin–ferroportin axis; although we did not notice a surge in hepcidin, we interestingly observed significantly low levels of ferroportin in TB patients, which further point towards iron sequestration in cellular stores (29–33). The reduced circulatory levels of iron carrier proteins like transferrin in ATB patients (**Figure 6B**) might be because of reduced circulatory iron levels (16). Higher transferrin receptor and low transferrin levels observed in ATB subjects (**Figure 6B** and **Figures S4–S8**) might enhance holotransferrin–transferrin receptor complex formation to deplete the circulatory iron and transferrin. Iron-sensing proteins like hemojuvelin and aconitase (IRP1) were found to be lower in ATB subjects (**Figures 6C**, **S4–S8**) (33, 34). Inflammation reduces hepatic hemojuvelin production and impacts iron sensing, which might be happening in these TB patients (**Figure 7B**) (35, 36). In addition, the iron transporters Nramp1 and Nramp2 (Dmt1) involved in cellular influx of iron were found to be reduced in TB patients’ serum samples compared to NTB and healthy controls. Nramp1 is involved in an evolutionary role to deplete the phagosomal iron by transporting phagosomal iron (Fe^2+^) to the cytosol; it may be an important host factor to starve the pathogen for iron. Decreased Nramp1 levels indicate that *Mtb* interferes with the host system to retain the iron in the phagosome to survive and thrive. Keeping in view the Nramp1 polymorphism, this can be an interesting aspect to explore nutritional immunity. Most of these iron-metabolizing proteins show a trend returning towards baseline after completion of a 6-month-long anti-TB treatment (**Figures 6A–D** and **S7**) and none of these TB drugs targeting on the host iron metabolism. It seems that although altered iron metabolism in TB patients is well known, its therapeutic potential is less explored. Although this study has a small population size, it provides detailed profiling of trace metals, cytokines, and abundance of iron-metabolizing proteins in an important study population. These findings, after careful validation in a larger population size in diverse clinical settings, will be useful to understand nutritional immunity and develop additional therapeutic strategies.

In conclusion, our study demonstrated a dysregulated iron homeostasis and suppressed Th2 profile. This research provides evidence for paradigms in nutrient metal homeostasis at the host–pathogen interface. The clinical benefits of selenium supplementation in TB patients need further validation. Additionally, the observed AI and dysregulated iron homeostasis in TB patients may provide a useful target for adjunct therapeutics development.

## Data availability statement

The raw data supporting the conclusions of this article will be made available by the authors, without undue reservation.

## Ethics statement

The studies involving human participants were reviewed and approved by Institutional Ethics Committee, AGMC, NHAK, AMC and ICGEB. Written informed consent to participate in this study was provided by the participants’ legal guardian/next of kin.

## Author contributions

SK and SuS carried out all the laboratory profiling experiments. Study subject recruitment for classification was conducted by HG, RD, SoS, RD, RK, WK, TD, and RB under the guidance of AT, ArD, BG, LS, VK, and AnD. Funds for this work were generated by RN, LS, VK, and AnD. AP shared resources and was involved in experimental planning. SK and RN wrote the first draft of the manuscript and revised it, incorporating the comments of all authors. All authors contributed to the article and approved the submitted version.
