# Supplementary material for: Low circulatory Fe and Se levels with a higher IL-6/IL-10 ratio provide nutritional immunity in tuberculosis
Source: Front Immunol. 2023 Jan 12;13:985538. doi: 10.3389/fimmu.2022.985538 (PMC9878310; doi:10.3389/fimmu.2022.985538)
Supplement: Supplementary file 1 [file DataSheet_1.pdf]

**Low circulatory Fe and Se levels with a higher IL-6/IL-10 ratio provide nutritional immunity in tuberculosis**

Sandeep R. Kaushik, Sukanya Sahu, Hritusree Guha, Sourav Saha, Ranjit Das, Rukuwe-u Kupa, Wetetsho Kapfo, Trinayan Deka, Rumi Basumatary, Asunu Thong, Arunabha Dasgupta, Bidhan Goswami, Amit Kumar Pandey, Lahari Saikia, Vinotsole Khamo, Anjan Das, Ranjan Kumar Nanda

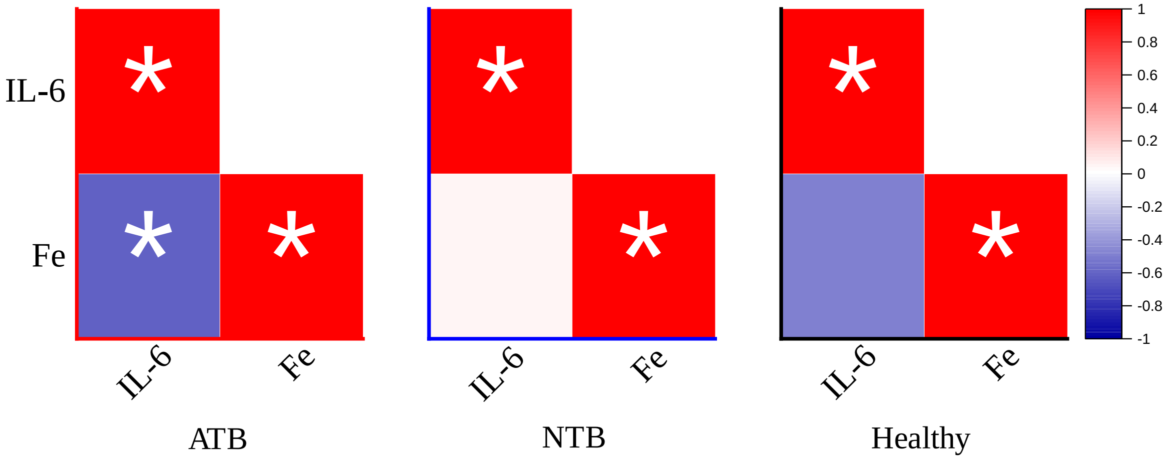


Supplementary Figure S1: Statistical significance for serum IL-6 vs Iron (Fe) correlation [Pearson (r)] in active tuberculosis patients (ATB) and controls (non-tuberculosis: NTB and healthy subjects). *:p<0.05.

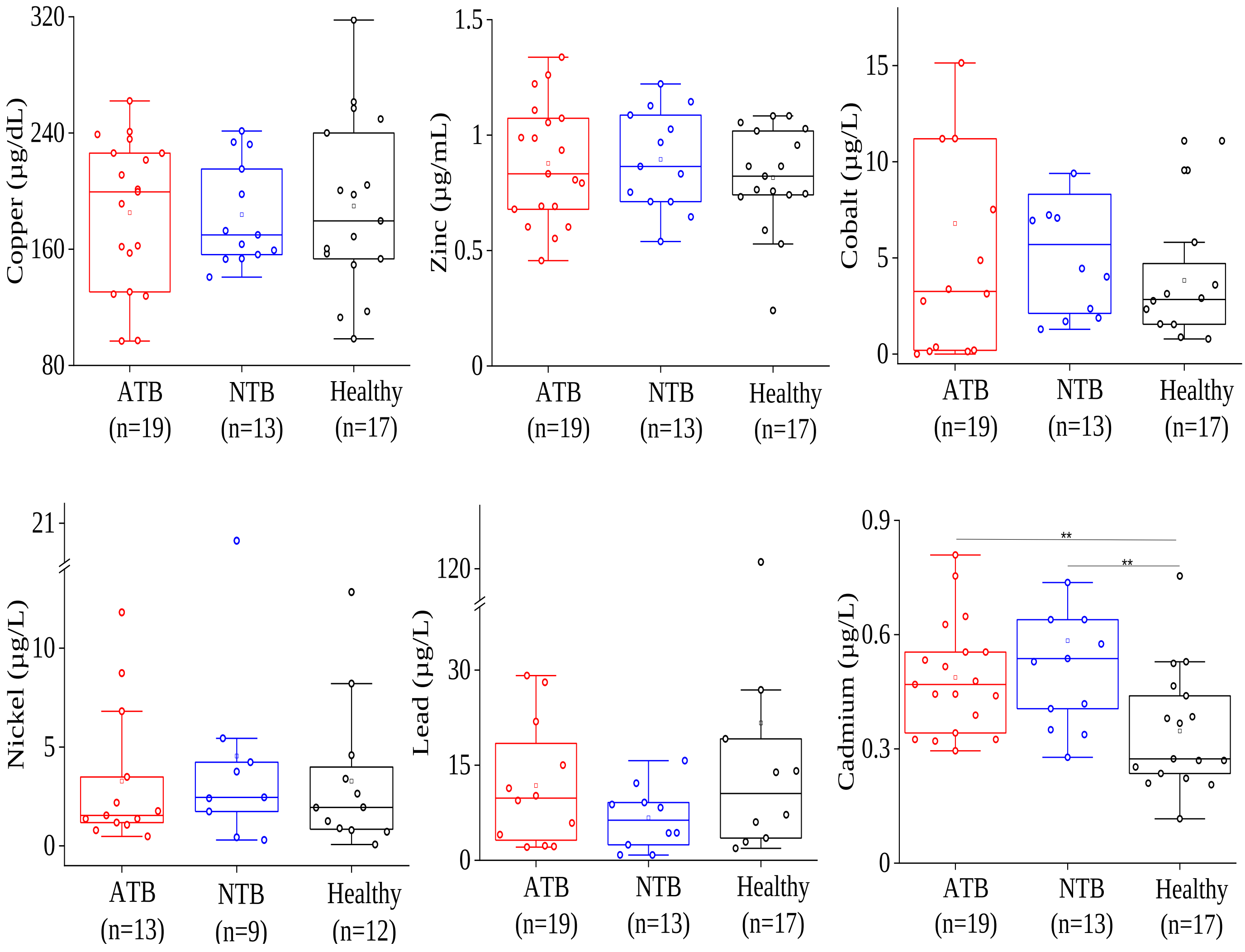


Supplementary Figure S2: Serum trace metal concentrations as estimated by Inductively coupled plasma mass spectrometry (ICP-MS) in active tuberculosis patients (ATB) and controls (non-tuberculosis: NTB and healthy subjects). **: $p<0.01$.

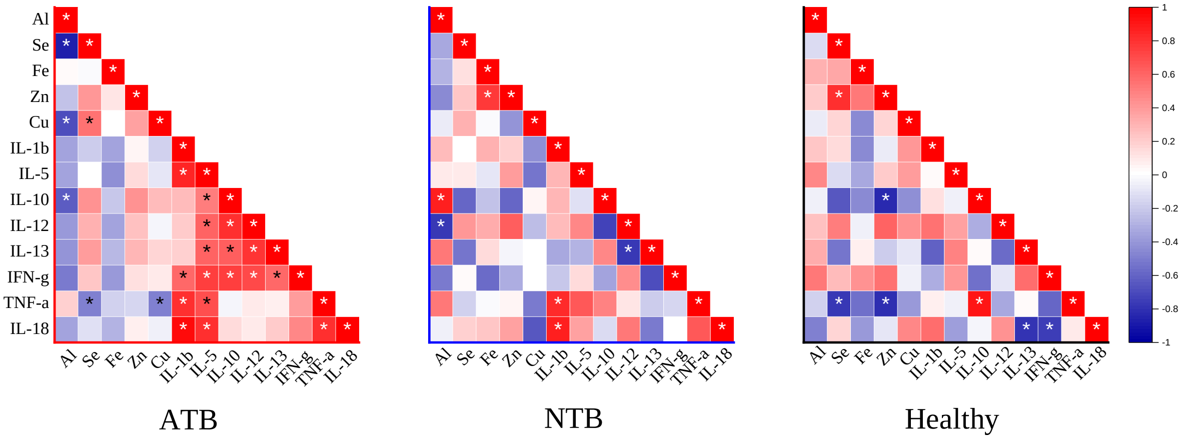


Supplementary Figure S3: Statistical significance for pairwise correlation [Pearson (r)] for important molecules in active tuberculosis patients (ATB) and controls (non-tuberculosis: NTB and healthy subjects). *:p<0.05.

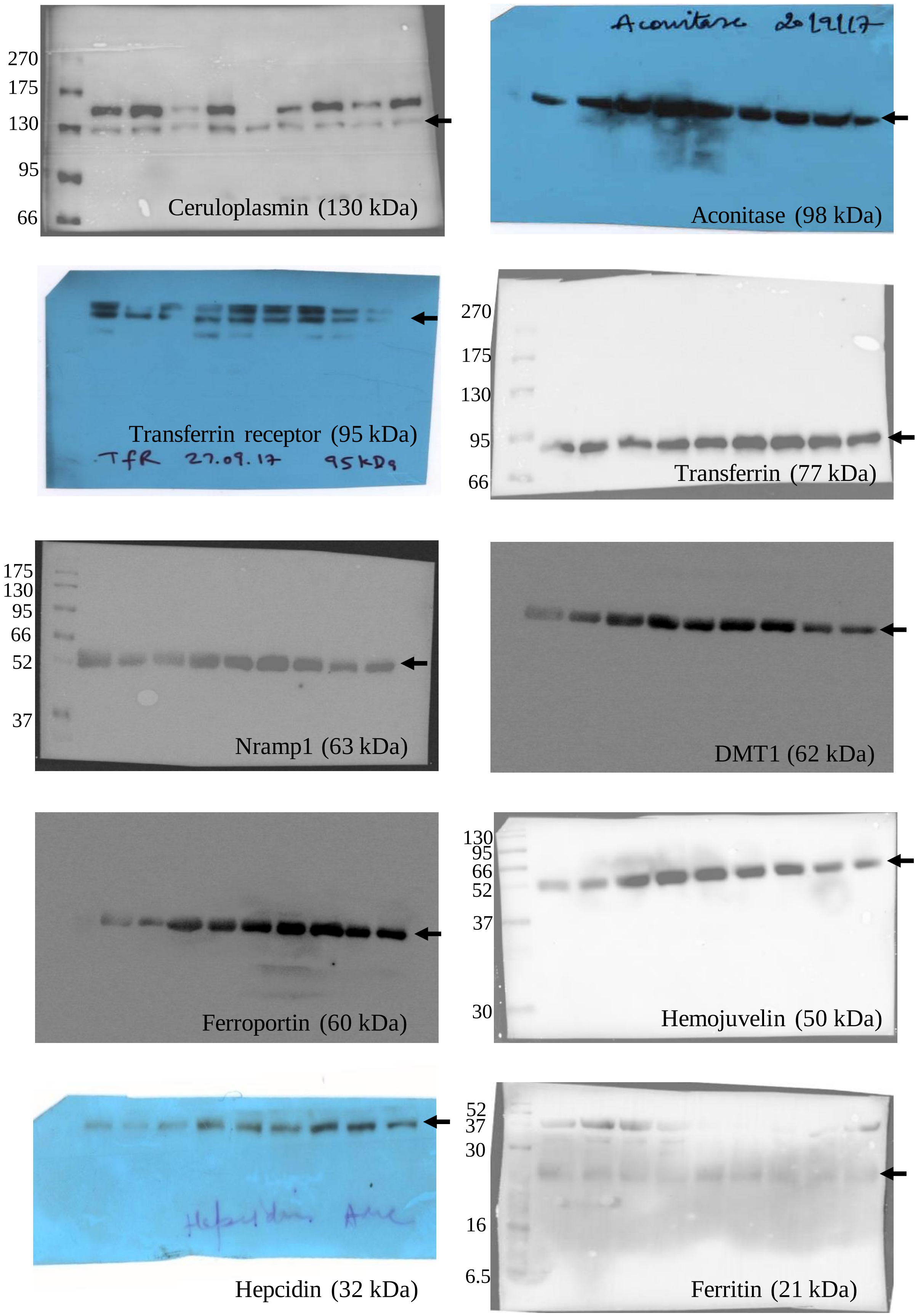


Supplementary Figure S4: Complete Western blot image of serum iron metabolizing proteins, used for intensity calculation in samples from active tuberculosis patients (ATB) and controls (non-tuberculosis: NTB and healthy subjects) and presented in Figure 2A and 2B. A representative parallel silver stained gel image is shared in Supplementary Figure S8A.

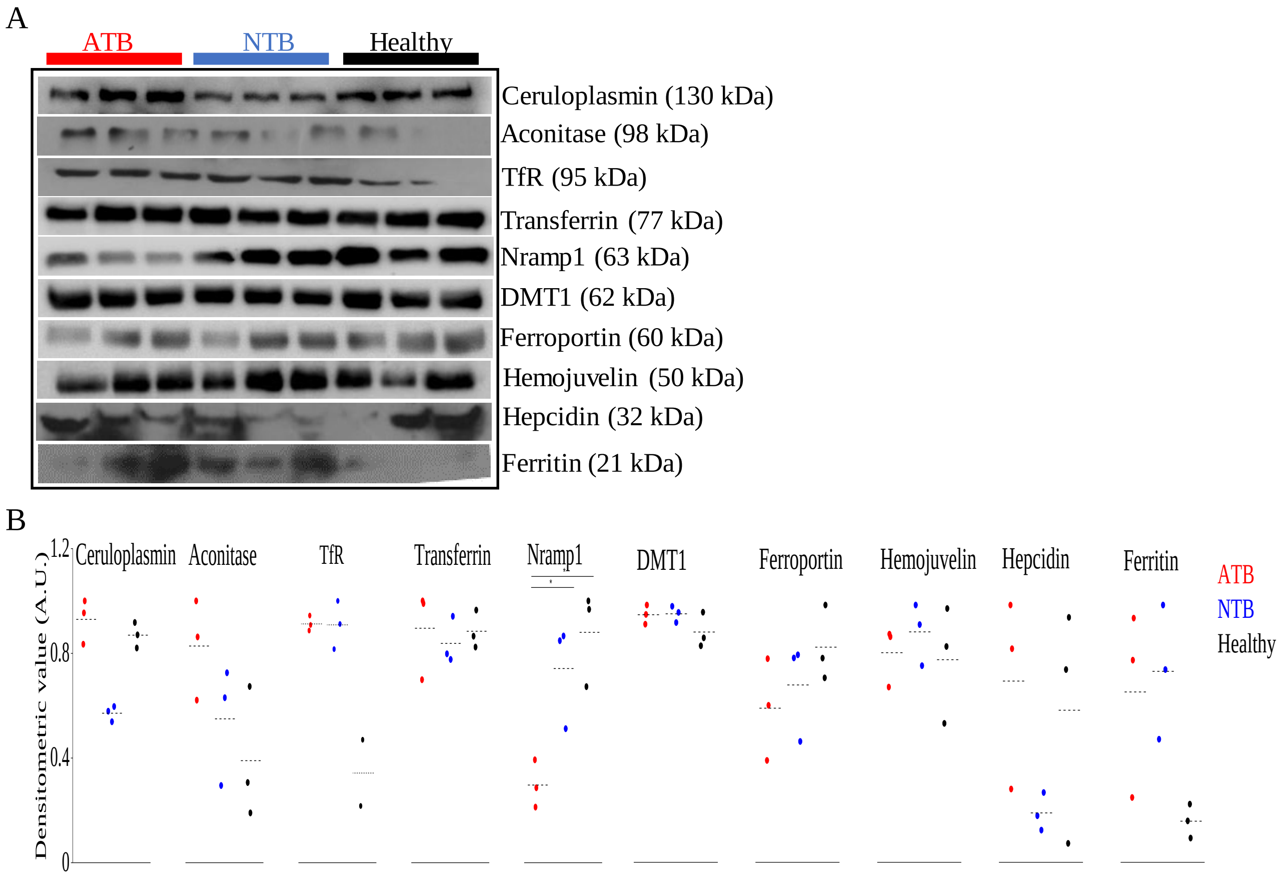


Supplementary Figure S5: Western blot images of serum iron metabolizing proteins in active tuberculosis patients (ATB) and controls (non-tuberculosis: NTB and healthy subjects) from 2nd clinical site (S5A). Complete blot images are presented in Supplementary Figure S7. Variation in intensities of individual serum protein levels in ATB, NTB and healthy control groups (S5B). TfR: Transferrin receptor; DMT1: Divalent metal ion transporter 1; *:p<0.05.

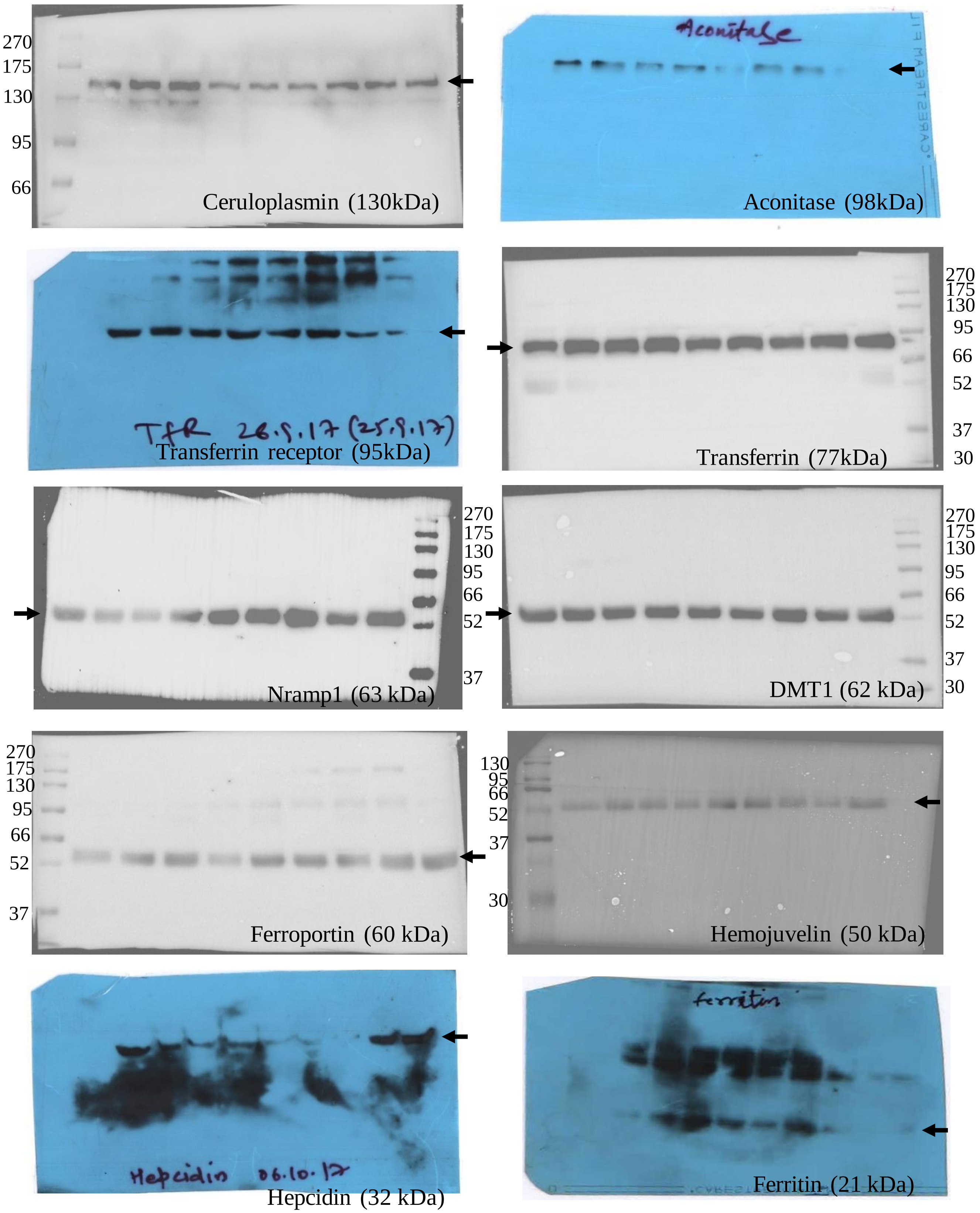


Supplementary Figure S6: Complete Western blot image of serum iron metabolizing proteins, used for intensity calculation in active tuberculosis patients (ATB) and controls (non-tuberculosis: NTB and healthy subjects) and presented in Supplementary Figure S5. A representative parallel silver stained gel image is shared in Supplementary Figure S8B.

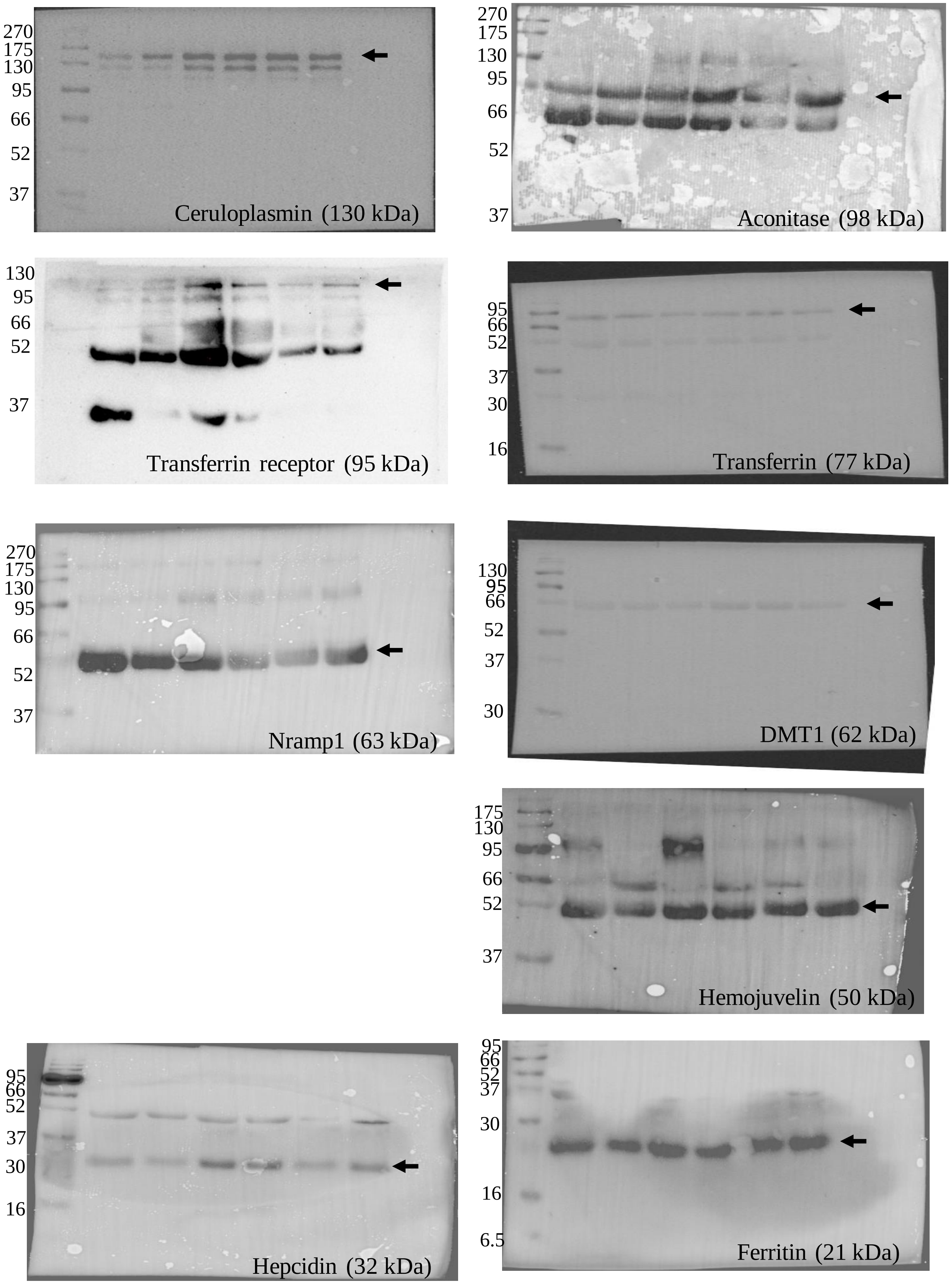


Supplementary Figure S7: Complete Western blot image of serum iron metabolizing proteins, used for intensity calculation in longitudinally followed up active tuberculosis patients (ATB) at time of presentation (0 month) and completion of treatment (6 months, clinically cured) and presented in Figure 2C and 2D. A representative parallel silver stained gel image is shared in Supplementary Figure S8C.

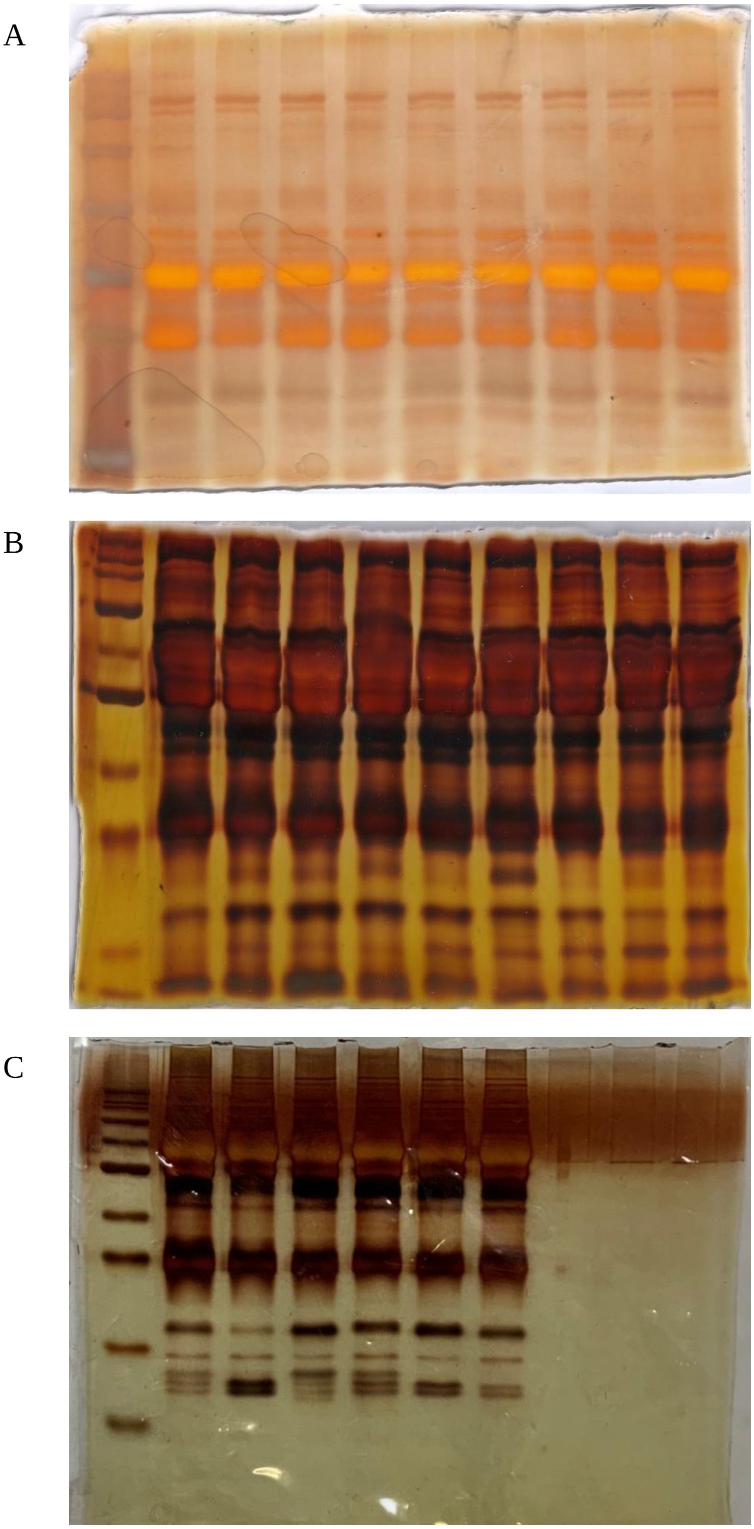


Supplementary Figure S8: Silver stained gel-images for loading control. A) Representative gel image for Supplementary Figure S5. B) Representative gel image for Supplementary Figure S6. C) Representative gel image for Supplementary Figure S7.
